# MMR Deficiency is Homogeneous in Pancreatic Carcinoma and Associated with High Density of Cd8-Positive Lymphocytes

**DOI:** 10.1245/s10434-020-08209-y

**Published:** 2020-02-27

**Authors:** Christoph Fraune, Eike Burandt, Ronald Simon, Claudia Hube-Magg, Georgia Makrypidi-Fraune, Martina Kluth, Franziska Büscheck, Doris Höflmayer, Niclas Ch. Blessin, Tim Mandelkow, Wenchao Li, Daniel Perez, Jakob R. Izbicki, Waldemar Wilczak, Guido Sauter, Jörg Schrader, Michael Neipp, Hamid Mofid, Thies Daniels, Christoph Isbert, Till S. Clauditz, Stefan Steurer

**Affiliations:** 1 Institute of Pathology, University Medical Center Hamburg-Eppendorf, Hamburg, Germany; 2 General, Visceral and Thoracic Surgery Department and Clinic, University Medical Center Hamburg-Eppendorf, Hamburg, Germany; 3 I. Medical Department – Gastroenterology and Hepatology, University Medical Center Hamburg-Eppendorf, Hamburg, Germany; 4 General, Vascular and Visceral Surgery Clinic, Itzehoe Medical Center, Itzehoe, Germany; 5 General, Visceral Thoracic and Vascular Surgery Clinic, Regio Clinic Pinneberg, Pinneberg, Germany; 6 General, Visceral and Tumor Sugery Clinic, Albertinen Hospital, Hamburg, Germany; 7 Department of General, Gastrointestinal and Colorectal Surgery, Amalie Sieveking Hospital, Hamburg, Germany

## Abstract

**Background:**

Microsatellite instability (MSI) has emerged as a predictive biomarker for immune checkpoint inhibitor therapy. Cancer heterogeneity represents a potential obstacle for the analysis of predicitive biomarkers. MSI has been reported in pancreatic cancer, but data on the possible extent of intratumoral heterogeneity are lacking.

**Methods:**

To study MSI heterogeneity in pancreatic cancer, a tissue microarray (TMA) comprising 597 tumors was screened by immunohistochemistry with antibodies for the mismatch repair (MMR) proteins MLH1, PMS2, MSH2, and MSH6.

**Results:**

In six suspicious cases, large section immunohistochemistry and microsatellite analysis (Bethesda panel) resulted in the identification of 4 (0.8%) validated MSI cases out of 480 interpretable pancreatic ductal adenocarcinomas. MSI was absent in 55 adenocarcinomas of the ampulla of Vater and 7 acinar cell carcinomas. MMR deficiency always involved MSH6 loss, in three cases with additional loss of MSH2 expression. Three cancers were MSI-high and one case with isolated MSH6 loss was MSS in PCR analysis. The analysis of 44 cancer-containing tumor blocks revealed that the loss of MMR protein expression was always homogeneous in affected tumors. Automated digital image analysis of CD8 immunostaining demonstrated markedly higher CD8 + tumor infiltrating lymphocytes in tumors with (mean = 685, median = 626) than without (mean = 227; median = 124) MMR deficiency (*p* < 0.0001), suggesting a role of MSI for immune response.

**Conclusions:**

Our data suggest that MSI occurs early in a small subset of ductal adenocarcinomas of the pancreas and that immunohistochemical MMR analysis on limited biopsy or cytology material may be sufficient to estimate MMR status of the entire cancer mass.

**Electronic supplementary material:**

The online version of this article (10.1245/s10434-020-08209-y) contains supplementary material, which is available to authorized users.

Despite recent progress in cancer therapy, pancreatic cancer—with more than 55,000 new cases diagnosed in the United States annually—still confers dismal prognosis.^1^ Due to the lack of early symptoms, late diagnosis, early metastatic dissemination, and ineffective systemic therapies, prognosis for these tumors is poor. Although pancreatectomy can prolong life in eligible patients, less than 5% of patients will survive more than 5 years. Immune checkpoint inhibitors have become a breakthrough treatment modality for many solid cancers but initially showed little effect in pancreatic cancer.^2,3^ However, based on data showing strong favorable responses of microsatellite instable (MSI) tumors—independent of the site of tumor origin—to the PD-1 antibody pembrolizumab, this drug obtained a site-agnostic FDA approval for treatment of advanced cancers with MMR deficiency/MSI-high.^4,5^ As several MSI pancreatic cancers had responded well to pembrolizumab in a recent study, the MSI status of pancreatic cancers is increasingly gaining interest.^6^

MSI reflects a hypermutator phenotype inducing a high mutational load in affected cancers and is typically caused by a deficient mismatch repair (MMR) system unable to resolve short slippage DNA errors that occur during cell cycle. It can be detected directly with polymerase chain reaction (PCR)-based methods demonstrating variable allele length of microsatellites or indirectly by identifying an expression loss of the MMR proteins MLH1, PMS2, MSH2, or MSH6 by immunohistochemistry (IHC). MSI occurs in various malignancies. Highest frequencies have been reported for endometrial (21–30%), colon (up to 19%), and stomach cancer (6–22%), but an increasing number of studies is showing that MSI can be found in virtually all individual cancer types at a frequency of approximately 1%.^7–15^ Tumor-infiltrating lymphocytes have been linked to tumor phenotype as well as favorable patient outcome or response to therapy in various tumor types.^16–18^ MSI is associated with a high number of tumor-infiltrating lymphocytes providing indirect evidence for a particular role of the antitumoral immune response in such tumors, presumably due to increased neoantigen production.^19–21^

Reports on MSI in pancreatic cancer described variable results. MSI has been reported to occur in 0–22% of unselected cases when IHC was used to assess MMR deficiency and in 0–17% in reports utilizing PCR based methods.^22–25^ Given the pivotal impact on the selection of treatment, precise assessment of MSI is important. Especially in those pancreatic cancers that are not amenable to surgery, therapeutically relevant molecular information is generally obtained from biopsies or cytological specimen. Such a small fraction of the primary tumor may not always be reflective of the molecular status of the entire cancer mass. Tumor heterogeneity can confound molecular diagnostics and diminish the success of targeted therapies. Intratumoral heterogeneity of MMR status has been described in some cases of colorectal and endometrial cancer but has so far not been analyzed in pancreatic adenocarcinoma.^26–30^

To learn more on MSI heterogeneity in pancreatic cancer, a cohort of 597 operated pancreatic cancers was screened by IHC for loss of the MMR proteins MLH1, PMS2, MSH2, and/or MSH6 on a tissue microarray (TMA). Cases with suspected MSI were further analyzed by PCR and repeated IHC on large sections followed by a thorough analysis of all available cancer-containing tissue blocks for possible intratumoral heterogeneity. Moreover, the relationship of MSI with the number of CD8-positive tumor-infiltrating lymphocytes was analyzed to assess the biologic impact of MSI in pancreatic cancers.

## Materials and Methods

### Tissue Microarray

A tissue microarray was constructed from a consecutive series of 597 pancreatic carcinomas treated by pancreatectomy (pT ≥ 2), of which the specimens were analyzed at the Institute of Pathology of the University Medical Center Hamburg-Eppendorf. The series included 529 pancreatic ductal adenocarcinomas, 61 adenocarcinomas of the ampulla of Vater, and 7 acinar cell carcinomas. Data on pT and pN category, histological grade, tumor diameter, and presence or absence of distant metastasis were taken from the pathology reports. TMA construction was done as described.^31^ In brief, tissue cylinders with a diameter of 0.6 mm each were taken from representative tumor areas of selected tumor “donor” tissue blocks using a homemade semiautomated precision instrument and brought into empty recipient paraffin blocks. Utilization of archived diagnostic leftover tissues for manufacturing of tissue microarrays and their analysis for research purposes as well as patient data analysis has been approved by local laws (HmbKHG, §12,1) and by the local ethics committee (Ethics commission Hamburg, WF-049/09). All work has been performed in compliance with the Helsinki Declaration.

### Immunohistochemical Analyses

Freshly taken TMA sections were used for MMR protein analysis in an automated immunostainer (Dako/Agilent Autostainer Link 48). Primary antibody specific for MLH1 (clone ES05, mouse), PMS2 (clone EP51, rabbit), MSH2 (clone FE11, mouse), and MSH6 (clone EP49, rabbit) (all Ready-to-Use, all from DAKO, Glostrup, Denmark) was applied for 20 min (MLH1, MSH2, MSH6) or 30 min (PMS2). A manual approach was used for CD8 immunostaining. Deparaffinized slides were exposed to a heat-induced antigen retrieval procedure for 5 min in an autoclave at 121 °C in pH 7,8 Tris–EDTA-Citrate buffer. Primary antibody specific for CD8 (Oncodianova, mouse monoclonal antibody, Clone TC8, 1:450) was applied at 37 °C for 60 min. Bound antibody was visualized using the EnVision Kit (Dako, Glostrup, Denmark) according to the manufacturer’s directions. Nuclear MMR protein staining intensity was scored as 0, 1 + , 2 + , or 3 + in cancer cells and the fraction (percentage) of stained tumor cells was also recorded for each tissue spot. In spots showing a negative (0) result for the tumor cells, presence (+) or absence (−) of nuclear staining in peritumoral stromal or inflammatory cells was additionally recorded as an internal control. For TMA spots with suspected MSI, IHC was repeated on a large section of the routinely archived tumor material. In case of confirmed MMR deficiency/MSI, all available archived tumor-containing blocks also were analyzed by IHC.

### PCR Analysis

For all cases with suspected MMR deficiency based on TMA screening, fluorescent PCR-based assay (MSI Analysis System; Promega, Madison, WI) was used to analyze five microsatellite loci, including the two mononucleotide repeats (BAT25, BAT26) and three dinucleotide repeats (D2S123, D5S346, and D17S250) of the “Bethesda-Panel.” Analysis was based on DNA extracted from tumor tissue that was dissected from a large section of the tumor block corresponding to the respective TMA spot and from nonneoplastic control tissue of the patient. Percentage of tumor cells was at least 50% within the tumor area analyzed. MSI-high was assigned when at least two of the five markers of the Bethesda-Panel showed instability (e.g., length variation compared to control tissue), and MSI-low was diagnosed if only one of the analyzed loci showed instability. All other cases were considered microsatellite stable (MSS).

### Quantification of CD8 Immunostaining

Digital images of stained slides were acquired using Leica’s Aperio VERSA 8 automated microscope. TMA spots were automatically identified and analyzed using the Image Scope 12.3.3 software package (Leica Microsystems; Wetzlar, Germany) according to the following procedure: every TMA slide was scanned at 40 × magnification. Digital images were segmented using the Image Scope brightfield TMA-Tool, and the segmentation was corrected manually. Two Aperio ePathology Image Analysis macros (Leica Microsystems) were adjusted to determine the number of CD8^+^ cells in each tissue spot and to measure the corresponding area of each tissue spot. The number of stained cells and the area in square millimeters of each individual spot was used to calculate the density of CD8^+^ stained cells/mm^2^ (number of cells per square mm). Schematic representation of the workflow to detect CD8 positive cell density is shown in supplementary Fig. 1.

### Statistics

Statistical calculations were performed with JPM 14 software (SAS Institute Inc., NC, USA) and R version 3.5.1 (The R foundation).^32^ Analysis of variance (ANOVA) was performed to search for associations between the density of CD8 positive cells and tumor phenotype as well as MSI status.

## Results

### TMA Screening

**Fig. 1:**
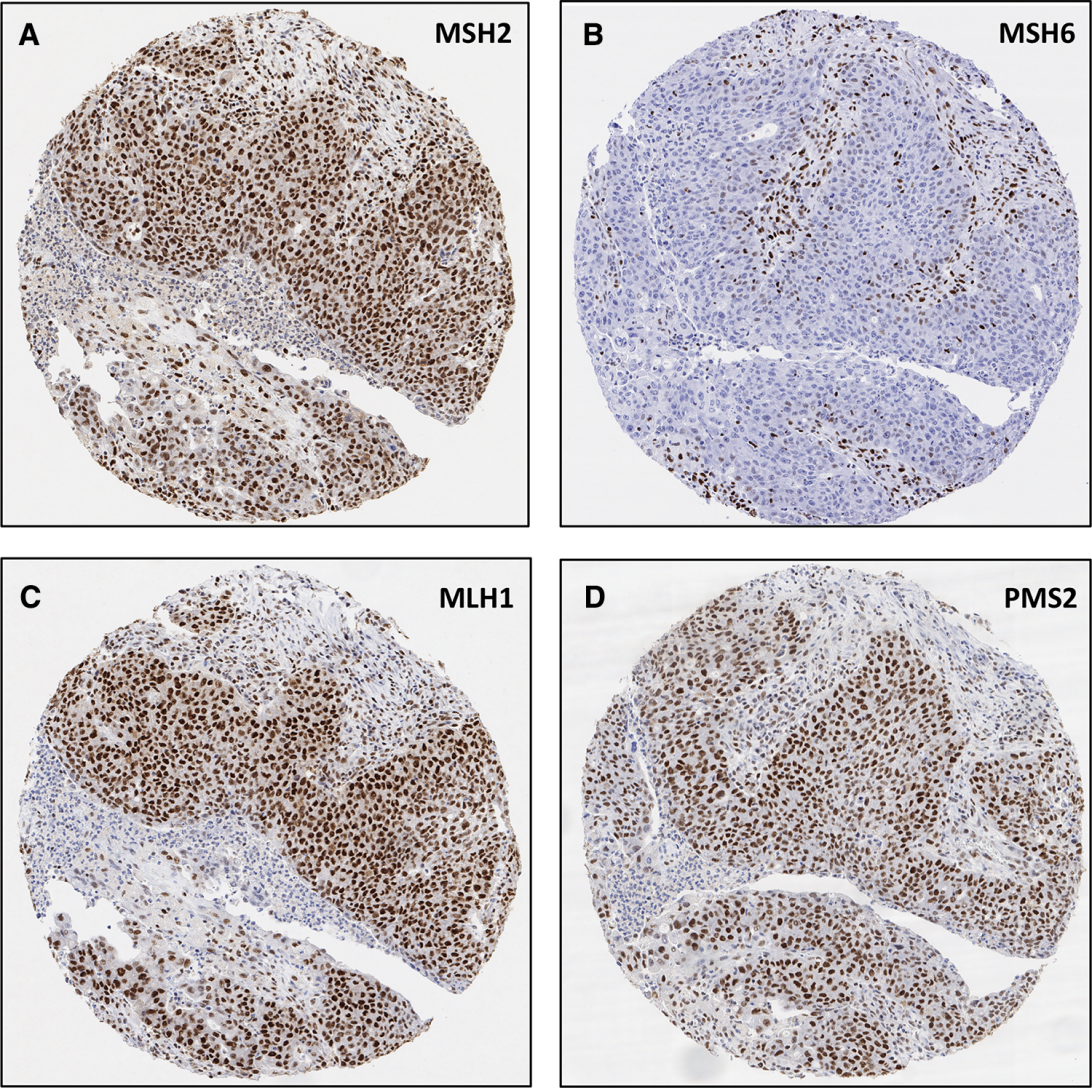
TMA spots of one pancreatic ductal adenocarcinoma with MSI associated with protein loss of MSH6 (**b**), whereas protein expression of MSH2 (**a**), MLH1 (**c**), and PMS2 (**d**) is retained. Original magnifications 15x, spot size 600 μm

A total of 542 of 597 (91%) cancers on the TMA were considered interpretable for MMR status, because either an unequivocal loss of staining of at least one of the four examined MMR proteins MLH1, PMS2, MSH2, or MSH6 was seen (MMR deficiency) or unequivocally retained expression of at least 3 MMR proteins without concomitant loss was observed (intact MMR status). Noninterpretable cancers (*n* = 55; 9%) were due to lack of unequivocal tumor on TMA spots or incomplete MMR results for tumor tissue, defined as retained expression but results available for ≤ 2 markers only. From the 542 interpretable cancers, 511 showed retained expression for all four MMR proteins (intact MMR status). In 25 cases, one MMR protein was not evaluable (due to lack of tissue or unequivocal tumor tissue on the spot) but the remaining 3 markers were unequivocally retained. These tumors were also considered to have an intact MMR status. For the remaining 6 cancers, the respective TMA spots showed loss of one or two MMR proteins with adequate positive control and were thus considered suspicious for MSI (Table 1). Representative micrographs from TMA screening are shown in Fig. 1.

**Table 1:** Summary of IHC and PCR data on pancreatic cancers with suspected MSI based on TMA screening. MMR evaluation by TMA screening is reported semiquantitatively by staining intensity (0–3) and percentage of positive tumor cells (0–100). In case of negativity (0) of tumor cells, internal control tissue was evaluated as positive (+) or negative (−)

Tumor type	TMA				Large section				Status IHC	Bethesda	tumor blocks	MMR pattern	CD8 density (CD8 + cells/mm^2^)
	MLH1	PMS2	MSH2	MSH6	MLH1	PMS2	MSH2	MSH6					
Ductal adenocarcinoma	2/70	2/100	0+	0+	pos	pos	neg	neg	MMR deficient	MSI-high (4/5)	15	homogeneous	55
Ductal adenocarcinoma	1/50	2/50	0+	0+	pos	pos	pos	pos (weak)	intact MMR	MSS (0/5)	–	–	37
Ductal adenocarcinoma	3/100	3/100	3/100	0+	pos	pos	pos	neg	MMR deficient	MSS (0/5)	16	homogeneous	958
Ductal adenocarcinoma	2/100	2/50	0+	0+	pos	pos	neg	neg	MMR deficient	MSI-high (5/5)	6	homogeneous	1434
Ductal adenocarcinoma	2/50	2/70	3/100	0+	pos	pos	pos	pos	intact MMR	MSS (0/4)	–	–	288
Ductal adenocarcinoma	2/100	3/100	1/30	0+	pos	pos	neg	neg	MMR deficient	MSI-high (4/5)	7	homogeneous	294

### MSI Validation and Heterogeneity Analysis

**Fig. 2:**
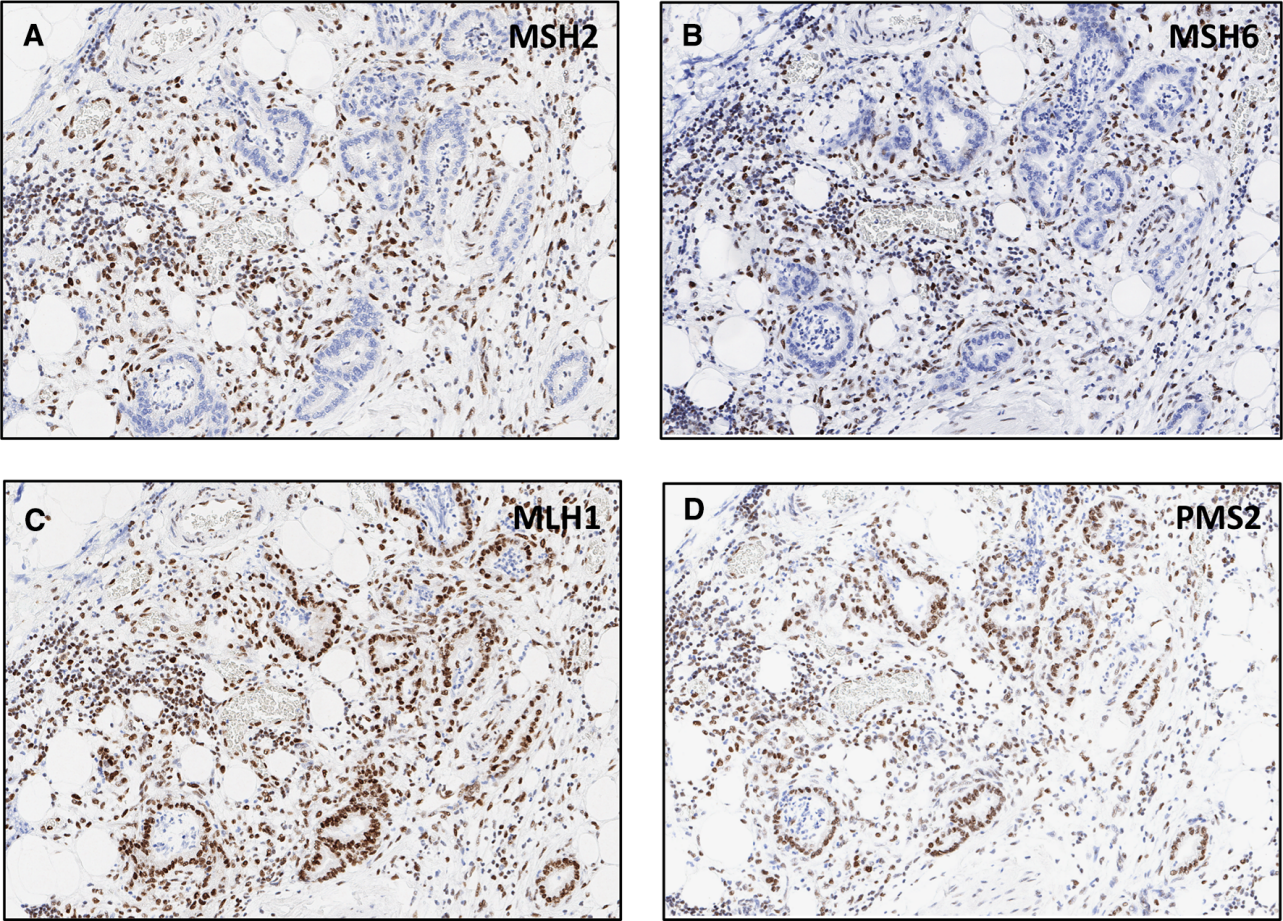
Protein loss of MSH2 (**a**) and MSH6 (**b**) on large sections of one pancreatic ductal adenocarcinoma. MLH1 (**c**) and PMS2 (**d**) protein expression is retained in the tumor cells. Staining in stromal cells and inflammatory cells is present as internal control. Original magnification 20x

Large section examination confirmed MMR deficiency in four of six suspected cases, all ductal adenocarcinomas, suggesting a prevalence for MSI in pancreatic ductal adenocarcinoma of 0.8% (4/480). Discrepant MMR status between TMA spots and large sections were always due to heterogeneous staining across the selected tumor blocks including areas where immunostaining was still weakly visible in some stromal cells but not visible in tumor cells. In these cases, the TMA cylinder had unluckily been taken from areas with markedly diminished immunoreactivity. All four confirmed MMR-deficient cases demonstrated MSH6 loss. MSH2 was additionally lost in three of these cases. Representative images from large sections are shown in Fig. 2. MSI analysis by PCR revealed MSI-high in three cancers and MSS in the cancer with isolated MSH6 loss (Table 1). A total of 44 large sections were analyzed from 4 patients (6–16 tumor blocks per patient) and revealed completely homogeneous MMR deficiency for each of the 4 identified MMR deficient cancers, including lymph node metastases that were present in 3 patients.

### Clinical Evaluation

Available clinical data revealed that additional cancers with MSI had occurred in two of the four patients with confirmed MMR deficient pancreatic cancer. One of these patients had a metachronous endometrioid endometrial cancer and the other patient a synchronous adenocarcinoma of the colon, both with MSH2 and MSH6 loss. In both cases, the expression loss involved the same MMR proteins as seen in the respective pancreatic cancer. For the other two patients with MMR-deficient pancreatic cancer, no clinical indications for Lynch Syndrome were found.

### Density of CD8-positive T Lymphocytes

**Fig. 3:**
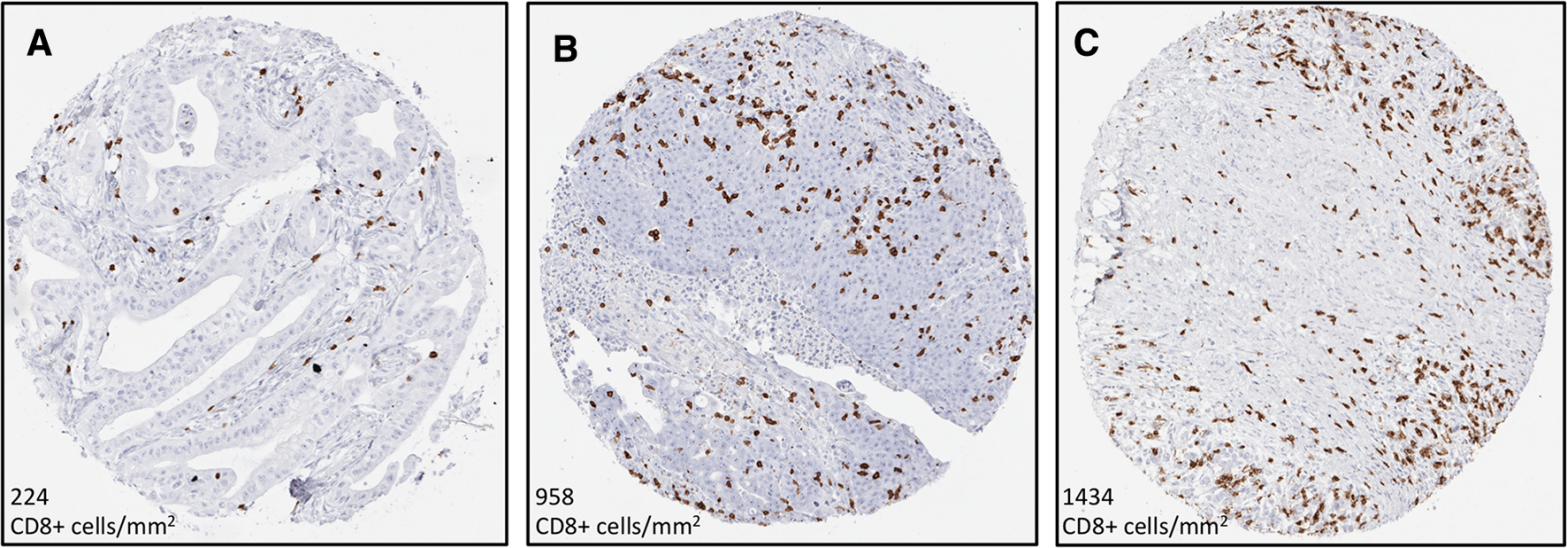
CD8-positive cell density in pancreatic ductal adenocarcinoma with intact MMR (**a**), with isolated protein loss of MSH6 associated with MSS in PCR-analysis (**b**), and with protein loss of MSH2 and MSH6 associated with MSI-high (**c**)

Density of CD8-positive cells could be evaluated in 551 of 597 cancers (92%) and varied widely from 0 to 2367 cells/mm^2^ in 551 interpretable cancers (median = 125; mean = 231). Representative images are shown in Fig. 3. The density of CD8-positive cells did not significantly vary between pancreatic ductal adenocarcinomas (*n* = 488; mean = 221, median = 122), adenocarcinomas of the ampulla of Vater (*n* = 54; mean = 301, median = 134), and acinar cell carcinomas (*n* = 7; mean = 256, median = 50; *p* = 0.17). The further comparison of the density of CD8-positive cells with tumor phenotype and MMR deficiency was limited to the ductal subset of pancreas cancers. A high density of CD8-positive cells was significantly associated with MMR deficiency. The density of CD8-positive cells was markedly higher in the 4 MMR-deficient cancers (mean = 685, median = 626) than in 520 cancers with intact MMR (mean = 227; median = 124; *p* < 0.0001). The cancer with unequivocal MMR defect (MSH6 loss) but MSS showed a particularly high CD8 density (958 cells/mm^2^; Table 1). The density of CD8 positive cells was statistically unrelated to pT, pN, M status, tumor grade (all supplementary Table 1), and tumor diameter (*p* = 0.4225, data not shown).

## Discussion

In this study we used a TMA for rapid screening for MMR deficiency in 597 carcinomas of the pancreas. Rare event detection is an ideal application for TMAs. Previous studies have found 42 tumors harboring IDH1 mutations by screening 15,531 prostate cancers (0.3%) or 43 tumors exhibiting CD117 overexpression in a cohort of 1654 breast carcinomas (2.6%).^33,34^ It is of note that only four of six cancers that were initially suspected to have MSI were confirmed in a subsequent large section validation. In both cancers that were not confirmed to exhibit MSI, the discrepancies were due to a staining gradient across the large slide, which is typically caused by inhomogeneous tissue fixation. In these cases, the tumor cells completely lacked MMR protein staining in the respective TMA spots while some weak staining was seen in stromal cells. This may reflect somewhat higher expression levels in a subset of stromal cells compared with the cancer cells in these cases.

Our rate of 0.8% of pancreatic ductal adenocarcinomas having MSI fits well with data from recent studies applying next generation sequencing. Salem et al. analyzed 7000 microsatellite loci in 870 pancreatic cancers by NGS and described 1.1% of tumors as MSI-high using NGS criteria.^35^ Hu et al. used a deep sequencing approach to evaluate microsatellites in all exons and selected introns of 341-468 cancer associated genes, followed by PCR and IHC validation and found MSI in 0.8% of pancreatic cancer.^36^ Findings were more variable in pure IHC studies and early PCR-based investigations using less stringent criteria to define MSI. Earlier IHC studies described MMR deficiency in 0–22%.^22,23^ Highest rates were reported in two TMA studies describing MMR deficiency in 15% of 265 and in 22% of 109 resected pancreatic cancers.^22,37^ Immunostaining issues in case of unevenly fixed tissues, as seen in our study, may serve as an explanation for such high frequencies of undetectable MMR protein in cancers. A large recent IHC-based analysis found MSI in 1.6% of 445 ductal pancreatic adenocarcinomas, which comes close to the ratio found by us.^38^ Earlier PCR-based studies on unselected cohorts of pancreatic adenocarcinomas described MSI in 0–17% of cases.^24,25^ The study with the highest fraction of positive cases found MSI in 8 of 46 cases, defining MSI as instability in at least 3 of 8 microsatellite loci analyzed.^25^ High rates of MSI (22%) also have been described in a selected cohort of 18 medullary pancreatic carcinomas.^39^

Evaluation of the MMR status throughout all available cancer-containing tumor blocks revealed homogeneous MMR protein loss throughout primary tumor and—if present—nodal metastases in all four ductal pancreatic adenocarcinomas with confirmed MSI. This fits with our previous observations of high homogeneity of MSI in prostate, bladder, ovarian, and neuroendocrine colorectal cancer (unpublished data).^40^ Overall, these data may suggest that MMR inactivation generally occurs early in tumorigenesis. Clinical history was suggestive of Lynch Syndrome in two of the four patients with MMR deficient pancreatic cancer as a metachronous MSI tumor with identical MMR pattern was reported in each case, presenting an explanation for the early MMR inactivation. Irrespective of its underlying cause (hereditary versus sporadic), homogeneity of MMR deficiency reduces the risk that microsatellite status obtained from small biopsies may not be representative for the entire cancer mass. Intratumoral heterogeneity is a potential strong confounder for individualized therapies, especially in pancreatic cancers that are not amenable to surgery.

Contemporary studies using state of the art technology usually reported MMR protein defects and MSI in about 0.5–3% of cancers, irrespective of the cancer type analyzed.^14^ Accordingly, the absence of detectable MMR protein loss in any of the 55 interpretable adenocarcinomas of the ampulla of Vater or 7 acinar cell carcinomas does not suggest absence of MSI in these tumor entities. In fact, MSI has previously been reported for both tumor entities in studies employing IHC. Agaram et al. found a MMR protein loss in 5 of 36 acinar cell carcinomas (14%), and Liu et al. reported MMR deficiency in 6 of 54 ampullary carcinomas (11%).^41,42^ Our results may suggest lower rates in these tumor types.

All four pancreatic ductal adenocarcinomas with MMR deficiency in our study exhibited protein loss of MSH6, which was accompanied by protein loss of MSH2 in three cancers and was isolated in one cancer. The functional role of MSH6 depends on heterodimerization with MSH2 (MutSalpha).^43^ Inactivation of MSH2, for instance due to proteasome-dependent degradation described for several disease-causing MSH2 mutations, results in concomitant MSH6 inactivation, because failure of heterodimerization causes rapid MSH6 degradation.^44,45^ In contrast, when MSH6 is inactivated, MSH2 is preserved due to increase of MSH3 by enhanced transcription and protein stability, facilitating alternative heterodimerization of MSH2 with MSH3 (MutSbeta).^46^ Accordingly, isolated MSH6 loss may be due to MSH6 inactivation while loss of both MSH6 and MSH2 may be attributable to MSH2 inactivation. Combined loss of MSH2 and MSH6 is rather infrequent in colorectal, endometrial, and gastric cancer, the tumor types with highest prevalence of MSI.^47,48^ In these entities, the vast majority of sporadic tumors with MSI are associated with MLH1 inactivation due to promotor hypermethylation. Consequently, MSI associated with loss of MSH2 and MSH6 is regarded as suspicious for a hereditary tumor origin in these cancer types.^49–51^ This may also hold true for a fraction of pancreatic cancers. In a recent NGS analysis of 53 hereditary pancreatic cancers without any known predisposition gene, one MSH2 and MSH6 germline mutation was described each.^52^ All 7 tumors with MSI among 833 pancreatic cancers (0.8%) identified in a recent study by Hu et al. using NGS sequencing were found to harbor germline mutations in the MMR genes.^36^ The concept of a possible germline involvement in pancreatic cancers with MSH6 and/or MSH2 expression loss is also supported by our findings. Two of our four patients with MSI pancreatic cancers had metachronous carcinomas with identical MMR protein loss.

It is of note that the tumor with an isolated but clear-cut loss of MSH6 expression was microsatellite stable in our PCR analysis. This is not surprising as a 100% concordance between MSI and MMR analysis is usually not found.^53,54^ Bartley et al. reported 13 discordant tumors among 591 colorectal carcinomas (2.2%).^53^ Discordance rate was even higher (5.5%) among 1.119 carcinomas from patients meeting clinical screening criteria (Amsterdam II or classical Bethesda guidelines) for Lynch Syndrome in a study by Engel et al. and Chapusot et al., which found 8 discordant cases among 100 sporadic right-sided colon cancers.^27,55^ Importantly, most discordant cases reported in the literature relate to MSI-high tumors lacking evidence for MMR deficiency by IHC.^53^ First, inactivating MMR mutations that escape detection by IHC—typically nonsense/missense mutations impairing protein function but retained antigenicity of the altered protein—may explain this.^55^ Second, the MMR system has much more players than MLH1, PMS2, MLH2, and MHS6. Inactivation of other MMR proteins, such as MSH3, PMS1, or EPCAM, may cause MSI without evidence of MMR deficiency by IHC. Discordant results characterized by IHC-detected MMR deficiency without MSI in PCR are generally rather rare but are well characterized for inactivating MSH6 mutations.^56^ MSH3 can partly compensate inactivated MLH6. However, in particular, MSH2/MSH3 heterodimers do not repair single base excisions as effectively as does the MSH2/MSH6 heterodimer.^57^ As the “Bethesda Panel”—used for defining MSI in this study—merely incorporates two mononucleotide repeats, MSI driven by MSH6 inactivation can be missed by PCR. In fact, no instability was found among any of the five repeat loci in the MSI tumor with isolated MSH6 loss in our study. Moreover, the “Bethesda Panel” (i.e., the selection of five mono- and dinucleotide repeats from the myriad of microsatellite loci throughout the genome) was developed based on data from MSI in colorectal cancers.^58^ Individual microsatellite loci may not be equally often affected by instability in different tissues and cell types, which relates to the observation that the likelihood for a frameshift depends on the transcriptional activity of the respective genomic region.^59,60^ Therefore, it is conceivable that the “Bethesda Panel” may not be universally suited for MSI detection across different tumor types.

Indirect support for true MMR deficiency in the identified MSS tumor with MSH6 protein loss comes from its strikingly high density of intratumoral CD8 positive lymphocytes (958 cells/mm^2^). The significant association between MMR deficiency and high number of intratumoral CD8-positive lymphocytes found for pancreatic cancer in this study is consistent with earlier data from colorectal, endometrial and stomach cancer.^19–21,61^ Elevated lymphocyte counts may represent evidence for immunogenic events having occurred in a cancer, a feature that is believed to be essential for successful therapy with immune checkpoint inhibitors. The significantly higher CD8 density in MMR deficient compared with MMR intact pancreatic cancers may thus provide an additional hint towards a potential utility of immune checkpoint inhibitors in pancreatic cancers as suggested by the successful treatment of several cancers in a site-agnostic clinical trial.^6^

Paralleling the advances in individual tumor therapy in modern oncology and the site-agnostic approval of recent drugs, there is a rapid increase of tumor testing in molecular pathology to identify “druggable” targets and a practical screening strategy to decide which tumor to test for certain alterations is desirable. Is it reasonable to test every pancreatic cancer for MMR deficiency and/or MSI, although the expected rate of MSI is “only” around 1%? In contrast to other widely performed predictive tests—like fluorescence in situ-hybridization for ROS1 or ALK fusion oncogenes in non-small cell lung cancer with reported frequencies only slightly higher (2–5%)—MMR IHC is fast and relatively inexpensive.^62^ Considering also the compelling response rates for immune checkpoint inhibitors in pancreatic cancers with MMR deficiency/MSI, including complete radiologic response in individual tumors, testing for MMR deficiency/MSI in pancreatic cancer may generally be considered and is encouraged in locally advanced/metastatic cancers by a recent NCCN guidelines update.^6,63^ Furthermore, detection of MMR deficiency/MSI can help to identify Lynch Syndrome patients and the evolving scientific interest in MSI has now made clear that MSI cancers due to germline mutations of MMR genes are not limited to colorectal and endometrial cancer as around half of all germline MMR mutations do occur in other tumor types, including pancreatic cancer.^15^ Parallel sequencing (NGS-based) methods to detect MSI have recently been developed, which may simplify molecular testing algorithms in cancer patients in the future, at least for tumor types where NGS is routinely applied to search for therapeutic targets.^64,65^

It is a limitation to this study that follow-up data were unavailable from our patients. The clinical impact of MSI and the density of CD8 positive cells thus cannot be finally judged based on our data. The absence of a significant association of the density of CD8-positive cells and pT, pN, M status, tumor grade, and tumor diameter does not suggest a pivotal clinical relevance of the CD8 density, at least in the absence of therapy regimens targeting the immune system. Several studies on cohorts from 86 to 214 patients with pancreatic cancer have, in contrast, suggested an association between the fraction of CD8 positive lymphocytes or neutrophils with tumor aggressiveness or clinical outcome of pancreatic cancer.^66–68^ However, spatial variance of CD8-positive cells—typically between central and peripheral tumor areas—is quite prevalent in pancreatic cancer and may confound analyses when information on the precise localization of the analyzed tumor area is not available.^66^ TMA-based studies—at least when TMA construction was not focused on tumor compartments as in our study—may underrepresent the invasive margin that has been shown to confer a prognostic role for CD8-positive cells in pancreatic cancer in particular.^68^ Furthermore, pancreatic cancer possess a unique microenvironment characterized by a dense desmoplastic stromal reaction that may enhance regional variability of lymphocytic density.^69^ For instance, CD8-positive cells at the invasive margin were significantly associated with overall survival in contrast to the tumor core.^70^ In that study, Tahkola et al. already used two TMA cores from each the tumor center and the invasive margin and only counted the higher score for statistical analyses to account for regional variation of CD8 positive cell density.^70^

## Conclusions

MSI identified by detecting MMR protein loss with IHC occurs in approximately 1% of pancreatic adenocarcinomas. The complete homogeneity seen in all four cancers with MSI suggests that MMR deficiency often is an early event in affected tumors. Small tissue probes as obtainable by biopsy or even aspiration cytology thus may be sufficient to determine a MSI status representative for the entire cancer mass.

## Electronic supplementary material

Below is the link to the electronic supplementary material.

Association of CD8 positive cell density with tumor phenotype in the subset of pancreatic ductal adenocarcinomas (XLSX 9 kb)

Schematic representation of the workflow to detect CD8 positive cells (blue) and to determine the area of the tissue spot (orange). Three examples of CD8 positive cell densities are given for tumors with intact MMR (A), with MSH6 protein loss associated with MSS in PCR-analysis (B), and with protein loss of MSH2 and MSH6 associated with MSI-high (C). Note that tissue spots can have a higher area as theoretically expected from a 0.6mm tissue punch. This is because the tissue expands when mounted on a slide (TIFF 18317 kb)
