# Correction to: MMR Deficiency is Homogeneous in Pancreatic Carcinoma and Associated with High Density of Cd8-Positive Lymphocytes

**DOI:** 10.1245/s10434-022-11798-5

**Published:** 2022-04-21

**Authors:** Christoph Fraune, Eike Burandt, Ronald Simon, Claudia Hube-Magg, Georgia Makrypidi-Fraune, Martina Kluth, Franziska Büscheck, Doris Höflmayer, Niclas Ch. Blessin, Tim Mandelkow, Wenchao Li, Daniel Perez, Jakob R. Izbicki, Waldemar Wilczak, Guido Sauter, Jörg Schrader, Michael Neipp, Hamid Mofid, Thies Daniels, Christoph Isbert, Till S. Clauditz, Stefan Steurer

**Affiliations:** 1 Institute of Pathology, University Medical Center Hamburg-Eppendorf, Hamburg, Germany; 2 General, Visceral and Thoracic Surgery Department and Clinic, University Medical Center Hamburg-Eppendorf, Hamburg, Germany; 3 I. Medical Department – Gastroenterology and Hepatology, University Medical Center Hamburg-Eppendorf, Hamburg, Germany; 4 General, Vascular and Visceral Surgery Clinic, Itzehoe Medical Center, Itzehoe, Germany; 5 General, Visceral Thoracic and Vascular Surgery Clinic, Regio Clinic Pinneberg, Pinneberg, Germany; 6 General, Visceral and Tumor Sugery Clinic, Albertinen Hospital, Hamburg, Germany; 7 Department of General, Gastrointestinal and Colorectal Surgery, Amalie Sieveking Hospital, Hamburg, Germany

## Correction to: Ann Surg Oncol (2020) 27:3997–4006 https://doi.org/10.1245/s10434-020-08209-y

The following acknowledgment was added to the original article:

Mrs. Devita Irene Putri contributed significantly to this study with results from her medical thesis.

