# Supplementary figures and images for: MMR Deficiency is Homogeneous in Pancreatic Carcinoma and Associated with High Density of Cd8-Positive Lymphocytes
Source: Ann Surg Oncol. 2020 Feb 27;27(10):3997–4006. doi: 10.1245/s10434-020-08209-y (PMC7471097; doi:10.1245/s10434-020-08209-y)

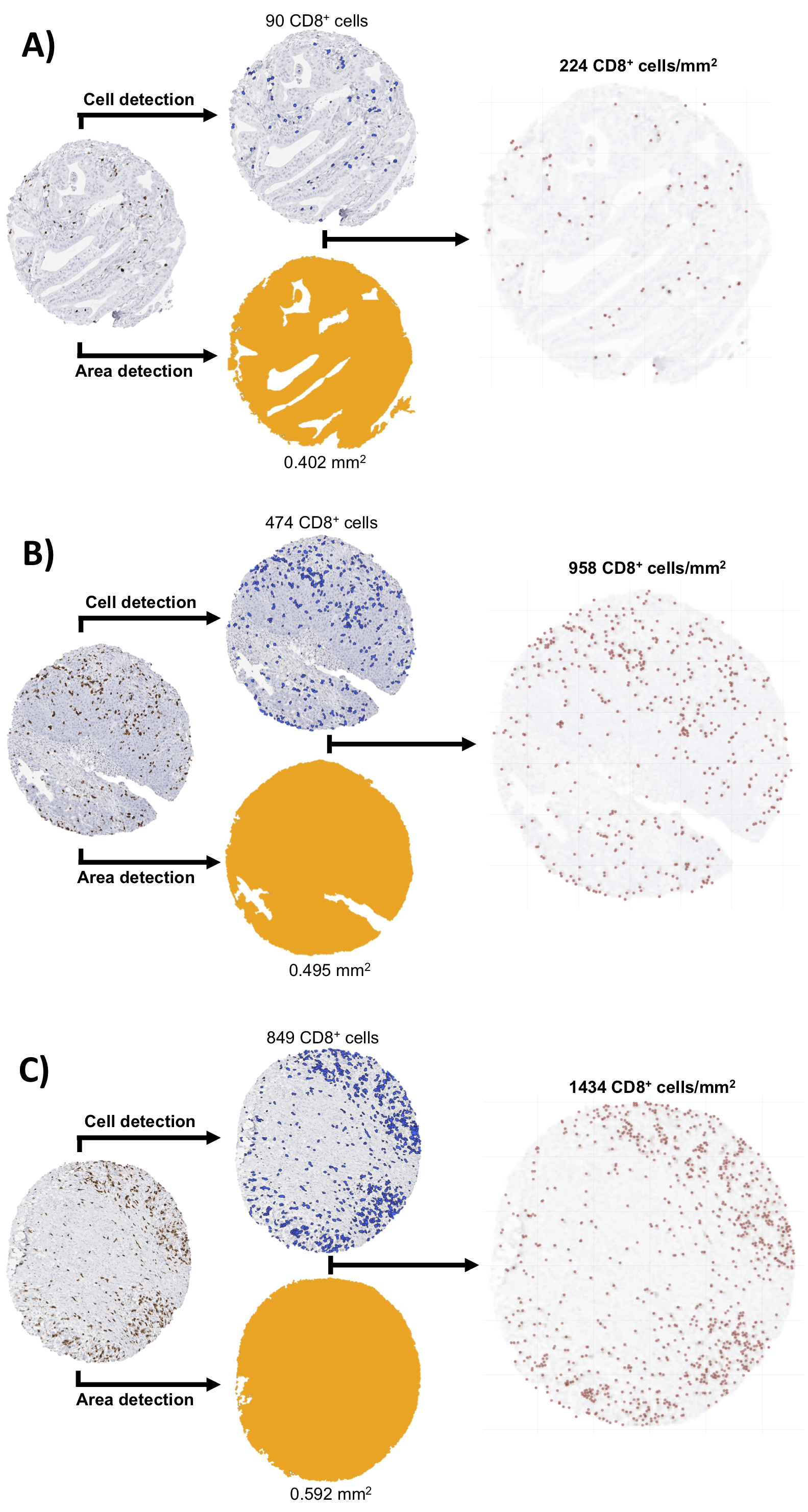

Supplement: Supplementary file 2 — Schematic representation of the workflow to detect CD8 positive cells (blue) and to determine the area of the tissue spot (orange). Three examples of CD8 positive cell densities are given for tumors with intact MMR (A), with MSH6 protein loss associated with MSS in PCR-analysis (B), and with protein loss of MSH2 and MSH6 associated with MSI-high (C). Note that tissue spots can have a higher area as theoretically expected from a 0.6mm tissue punch. This is because the tissue expands when mounted on a slide (TIFF 18317 kb) [file 10434_2020_8209_MOESM2_ESM.tiff]
